# University social responsibility (USR) in the context of Peruvian society: A systematic review

**DOI:** 10.12688/f1000research.138153.2

**Published:** 2025-07-21

**Authors:** Oscar Arbieto Mamani, Miguel Gerardo Mendoza Vargas, Rosmery Sabina Pozo Enciso, Percy Fritz Puga Peña, Teresa Villafuerte Palomino, Willie Alvarez Chavez

**Affiliations:** 1 Universidad Nacional Micaela Bastidas de Apurímac, Abancay, Peru; 2 Universidad La Salle, Arequipa, Peru

**Keywords:** University law; university social responsibility; Peru; systematic review; journal; Prisma; Scopus; Redalyc; Scielo

## Abstract

**Background:**

The university social responsibility (USR) goes beyond the traditional extension and solidary social projection of the universities, but the professional must improve and develop proposals in improvement of the country. The objective is to carry out a systematic review of the scientific production related to USR in Peru and to analyse the most important findings of this production.

**Method:**

A systematic review of articles in English and Spanish in Scopus, Redalyc, and SciELO was carried out, searching for research related to USR in the Peruvian context. Articles on USR in Latin America or the world were excluded. The search and filtering of articles was carried out until February 2023. Several filters were applied, starting with the search for titles according to the search equation. Then, articles that did not deal with USR were eliminated. Subsequently, abstracts were read and those that did not meet the inclusion criteria were discarded. Finally, the remaining research was analysed to obtain the necessary information for the research.

**Results:**

A total of 20 articles were analysed. The main results showed that university social responsibility in the Peruvian context seeks to benefit society and to form ethical and responsible students. However, more policies and actions are needed to encourage the participation of all universities in USR. It was found that 65% of the literature had a quantitative approach, 30% was qualitative and only 5% was mixed.

**Conclusion:**

University Social Responsibility (USR) seeks to benefit society, being students the key actors to improve their country with the professional development acquired in higher institutions. The implication for future research is to carry out more studies on USR but within the national university centres in the highlands and jungle areas of Peru, where it is possible to show the state of this topic in other areas of Peru.

## Introduction

In recent years, the production of goods and services has grown rapidly, and although it has allowed for the development of emerging economies, the increase in globalization and the advancement of the media has also led to neglect of labor rights, disregard for the environment, and rising levels of poverty in different countries around the world ( Comisión Económica para América Latina y el Caribe, 2018).

Due to the lack of attention to different stakeholder groups, the concept of corporate social responsibility has gained importance in the last century ( Palacio, 2020). The initial approach to corporate social responsibility was introduced by Bowen (1953), who stated that social responsibility is “the obligation of businessmen to pursue policies, to make decisions or to follow lines of action that are desirable in terms of the objectives and values of society” (p.44). All decisions made by entrepreneurs in various organizations have an impact on the rest of society ( Tiep, Huan, and Hong, 2021).

As important as understanding the components of social responsibility is measuring and implementing them. However, due to the diversity of organizations and their multiple characteristics, there are various methods used in the implementation of corporate social responsibility programs, and consequently, the results vary ( Barragán *et al., *2019, 2022).

While it is important to understand the implementation and results of this vision in different sectors and types of organizations, there are activities and institutions on which social pressures increase, and it is important to emphasize them given their nature and mission ( Garbanzo, 2015). This is the case with Higher Education Institutions and Universities, which have a high responsibility towards society as they are responsible for preparing the professionals of the future ( Santana, 2022).

It should be remembered that the mission of the university and higher education institutions is based on social awareness, as the university, above all, shapes individuals with all that entails culture, ethics, social commitment, and politics ( Ruiz and López, 2019). This is because it seeks to build knowledge and fulfil its missions of “education, research, and social dimension” ( Beltrán *et al.*, 2014, p.5).

As evident, the link between the university and society as a principle of responsibility has always existed. This is why university social responsibility (USR) is defined as a set of integral management policies that contribute to the challenges of the 21st century ( Ibarra and Fonseca, 2020), aiming to develop a series of actions that improve the university’s relationships with society and fulfil its responsibility towards all stakeholders (administrative staff, professors, students, and administrative personnel, among others) ( Medina *et al., *2017).

USR goes beyond traditional solidary social extension and outreach; it is not only about voluntary initiatives of assistance and service to vulnerable groups. It is important to coordinate university actors to transmit responsible knowledge, as it ensures the contribution of competencies and capabilities, which in turn leads to sustainable development and the education of future comprehensive professionals, as stated by Prieto *et al.* (2015), Cabrejos (2017), and Vallaeys and Álvarez (2019).

The rapid evolution of higher education and its increasing engagement with social development have highlighted the critical role of universities in fostering USR. Prior studies have examined the legal and ethical foundations of USR, emphasizing its significance in shaping socially responsible professionals. However, the methodological rigor of these studies has varied, necessitating a deeper evaluation of their quality and impact. This review integrates methodological assessments, policy implications, and diverse perspectives to offer a comprehensive understanding of USR in the Peruvian context.

The objective of this study was to conduct a systematic review of scientific production related to university social responsibility (USR) in Peru and analyze the most important findings from that production. To carry out the research, a systematic literature review (SLR) was conducted using three databases: Scopus, Redalyc, and SciELO, which were selected based on the degree of result updates they provide and the bibliometric analysis tools they include.

## Methods

For the development of the research, a search for scientific production associated with the topic of USR was conducted through a systematic review of articles. This method aims to identify, interpret, and evaluate different works carried out by scholars on a specific topic or field, based on the text ( Pardal and Ochoa, 2017) with the intention to “provide researchers and readers with clarifying information on a particular subject” ( Pardal and Pardal, 2020, p.1).

The systematic review of scientific literature was carried out based on the adaptation of the PRISMA (Preferred Reporting Items for Systematic Reviews and Meta-Analyses) methodology, a method published in 2009. It was designed to assist authors in conducting transparent systematic reviews, documenting the rationale for conducting a review, what the authors investigated and developed, and what their main findings were, based on the text by Page *et al. *(2021). Based on this, the research question for the review was formulated: How is university social responsibility developed in Peru?

As previously indicated, this study follows the PRISMA framework, ensuring a transparent and replicable systematic review. Articles were selected based on predefined inclusion criteria, including relevance to USR and empirical contributions within Peru. To enhance methodological clarity, this study incorporates a detailed assessment of the research designs, validity measures, and potential biases present in the selected articles. Furthermore, bibliometric analysis tools were employed to categorize and compare findings systematically.

### Database search

The search was conducted within the databases of Scopus, SciELO, and Redalyc, as they are among the most important repositories of journals and scientific production in Latin America and the Caribbean, based on the text by Miguel (2011).

The obtained results were refined after an analysis of titles and keywords, and those documents with titles containing the term ‘responsabilidad social universitaria (USR)’ and ‘Peru’ were used, both in Spanish and English in all three databases. The search for articles was conducted from December 9, 2022, until the last screening on February 5, 2023.

The equation used for the first search within the three databases only used: ‘Responsabilidad social universitaria’ and ‘university social responsibility’ ( Table 1).

**Table 1.** First systemic database search by study topic: results by equation.

Database	Search equation	Articles found
Scopus	‘Responsabilidad social universitaria’	84
	‘University social responsibility’	301
Scielo	‘Responsabilidad social universitaria’	109
	‘University social responsibility’	102
Redalyc	‘Responsabilidad social universitaria’	763
	‘University social responsibility’	185
Total		1,544

Then, in order to identify the articles located in the Peruvian reality, a second filter equation was applied.: AND ‘Perú’ ( Table 2).

**Table 2.** Second search in databases by study topic: results by equation.

Database	Search equation	Articles found
Scopus	‘Responsabilidad social universitaria’ AND ‘Perú’	12
	‘University social responsibility’ AND ‘Perú’	12
Scielo	‘Responsabilidad social universitaria’ AND ‘Perú’	4
	‘University social responsibility’ AND’Perú’	11
Redalyc	‘Responsabilidad social universitaria’ AND ‘Perú’	191
	‘University social responsibility’ AND ‘Perú’	53
Total		283

**Figure 1.**
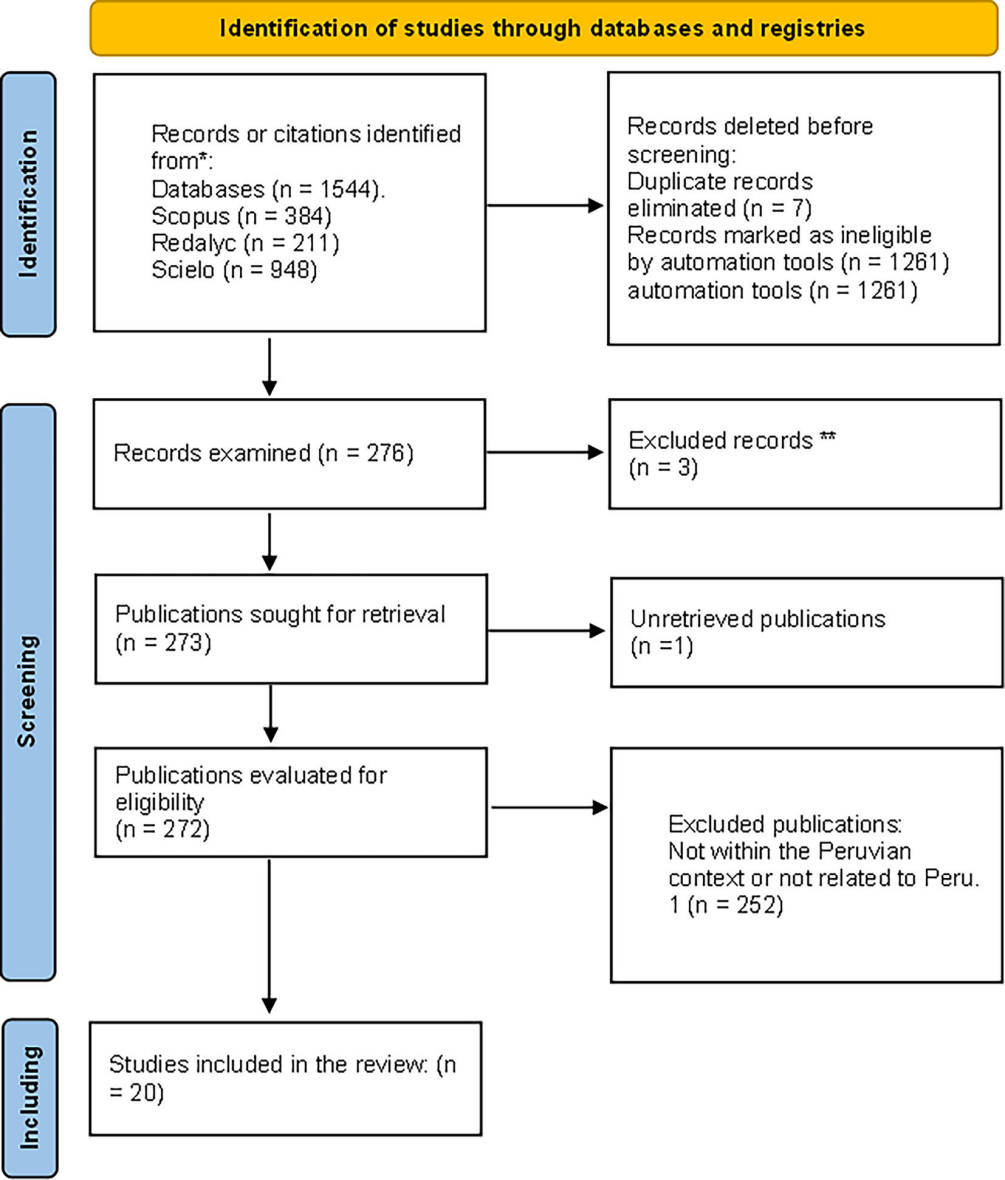
Flowchart of filtered article selection. Filtered articles from Scopus, Redalyc, and Scielo repositories.

As a final filter, all of the included search results were classified and the inclusion and exclusion criteria were applied to them ( Figure 1).

Inclusion criteria:

• Articles that have university social responsibility as a premise.
• Articles that have been developed in the Peruvian context.
• Empirical studies, both quantitative and qualitative, investigating aspects related to university social responsibility should be included.
• The studies can include key participants such as students, professors, administrative staff, university authorities, and other stakeholders related to university social responsibility, as well as literature review in the Peruvian context.

Exclusion criteria:

• Articles that are within the context of Latin America or the world.

The process for deciding whether the article met the criteria was first to review the titles of each research paper; where it was assessed whether the article met the university social responsibility theme. After the first filter, we proceeded to read all the abstracts and evaluate whether they were written within the Peruvian context. Finally, the total content of the manuscript was reviewed for the completion of the article. The filtering was performed by two of the authors and the examination of each record and each report retrieved was performed by each author, it should be noted that the distribution was done equally according to the number of articles between the number of authors. The review and analysis of each manuscript was carried out independently, but at the end a final compilation was made, which was done jointly by all authors.

Each author explored the contents of each manuscript in depth, meticulously evaluating the variable ‘university social responsibility’ in the context of the research. Crucial aspects such as the methodology employed, data collected and initial interpretations were considered. The individual analysis allowed a detailed exploration of each manuscript, always leaving diverse contributions from the perspective and experience in the interpretation of the data.

However, to ensure consistency and integration of the different analyses, a joint review phase was carried out, where the different approaches and results obtained were collated and contrasted. Constructive discussions and debates were encouraged to resolve discrepancies and ensure that the final interpretation was sound and substantiated. The process culminated in the creation of a final compilation reflecting the results and conclusions reached by consensus from the combination of the individual analyses.

## Results

The analysis of 20 selected articles reveals that 65% adopted a quantitative approach, 30% were qualitative, and 5% applied mixed methods. A critical assessment of these studies indicates that while many focus on student and faculty perceptions of USR, fewer explore its institutional integration and long-term effects. Additionally, by incorporating diverse perspectives, this study highlights contrasting viewpoints on the effectiveness of USR policies, ensuring a balanced discussion of its benefits and limitations ( Table 3).

**Table 3.** Articles studied through systematic review ( Arbieto *et al.*, 2023).

N°	Code	Author(s)	Title	Characteristics	Results
1	AC1	Martí, J., Pérez, G., y Cano, E. (2019)	Legislación de la responsabilidad social universitaria: Estudio de casos en universidades de Perú y Ecuador *[Legislation of university social responsibility: A case study of universities in Peru and Ecuador*]	Qualitative Documentary Analysis study, the author examined and analyzed “Ley universitaria 33733” (Perú) and “LOES, 2010” (Ecuador)	Higher education policies in Peru and Ecuador share approaches to university social responsibility (USR) and accreditation. However, they differ in aspects such as the integration of USR in institutions. Both countries prioritize the responsibility of universities towards the community, but with different approaches in terms of indicators and accountability.
2	AC2	Martí, J., Ignacio, A., y Fernández, A. (2018)	La responsabilidad social universitaria en Iberoamérica: análisis de las legislaciones de Brasil, España y Perú [ *University social responsibility in Ibero-America: analysis of the legislation of Brazil, Spain and Peru*]	Documentary analysis qualitative study Ley universitaria 33733 (Perú) Ley Orgánica 4/2007 (España) Ley 10 861 (Brasil)	In Brazil and Peru, USR is regulated as part of university management, focusing on outreach and community service activities, with evaluation indicators. On the other hand, Spain and the European Union link USR to a university oriented to economic development and to compete in the educational market.
3	AC3	Villegas, P., Cairampoma, A. (2021)	La responsabilidad social universitaria desde el derecho administrativo *[University social responsibility from an administrative law perspective]*	Qualitative study of documentary analysis of the regulations of Pontificia Universidad Católica del Perú.	This article highlights the role of the state and other actors in social responsibility, including the university. The Pontificia Universidad Católica del Perú focuses on university social responsibility (USR) as a requirement for licensing, adopting internal and external actions.
4	AC4	Pumacayo, I., Calla, K, y Yangali, J. (2020)	Responsabilidad social universitaria y la calidad de servicio administrativo *[University social responsibility and the quality of administrative services]*	Quantitative cross-sectional study with a sample of 268 students who were submitted to a questionnaire.	It found a significant relationship between university social responsibility and the quality of administrative service. The higher the university's social responsibility, the higher the quality of administrative service.
5	AC5	Vallaeys, F., y Álvarez, J. (2019)	Hacia una definición Latinoamericana de responsabilidad social universitaria. aproximación a las preferencias conceptuales de los universitarios *[Towards a Latin American definition of university social responsibility: an approach to the conceptual preferences of university students*]	Quantitative study that analyzed 740 people from several universities	The importance of impact management is highlighted and a Latin American definition of University Social Responsibility based on participation in territorial development and the management of administrative and academic impacts is proposed.
6	AC6	Zolezzi, L. (2010)	La responsabilidad social en la formación de los abogados *[Social responsibility in the training of lawyers]*	Qualitative study and analysis of law students' conception of USR in Peruvian universities.	Private universities tend to focus on business education, leaving aside social issues. Therefore, it is crucial that law schools carefully define their mission and align their processes with university social responsibility.
7	AC7	Saquicoray, P., y Cuadros, V. (2015)	Impacto de la responsabilidad social universitaria de la UNHEVAL en el desarrollo multisectorial de la provincia de Huánuco 2010-2013 *[Impact of UNHEVAL’s university social responsibility on the multi-sectoral development of the province of Huanuco 2010-2013]*	Qualitative documentary analysis study with a sample of 14 regional directorates of the study site.	The university extension and social projection work carried out is only a fulfillment of the statutory function, without going beyond the limits of the university.
8	AC8	Ortiz, I. (2010)	Proyección Social de Derecho: PROSODE, un aporte de proyección y responsabilidad social *[Social Projection of Law: PROSODE, a contribution to social projection and social responsibility]*	A qualitative study analyzed the PROSODE course taught at a Peruvian university.	The contribution of PROSODE of the PUCP Law School, experience of social projection and responsibility applied in the community, its contribution and projections.
9	AC9	Kovács, I. (2016)	Las relaciones públicas y la responsabilidad social de las universidades peruanas según la nueva ley universitaria *[Public relations and social responsibility of Peruvian universities under the new university law the new university law]*	A qualitative study analyzed USR according to university law.	University Social Responsibility ceased to be a simple charitable aid for the most deprived sectors of society.
10	AC10	Vargas, J. (2021)	Innovación social: ¿Nueva cara de la responsabilidad social? conceptualización crítica desde la perspectiva universitaria *[Social innovation: New face of social responsibility? Critical conceptualization from a university perspective]*	Qualitative study of documentary review and review of various manuscripts.	The term "social innovation" is fuzzy and ambiguous, but it is accepted that it involves addressing unmet social and environmental needs and improving the quality of life. The university plays a key role in this process through research, providing proactive and lasting solutions.
11	AC11	Ruiz, I., (2018)	Beca docente en el Perú: una experiencia de colaboración público-privada, más allá de la responsabilidad social universitaria *[Teaching scholarship in Peru: an experience of public-private collaboration, beyond university social responsibility]*	Documentary analysis study	To this end, efforts and resources were allocated beyond the stipulations of the framework agreements signed, assuming the mission entrusted as a process of university social responsibility (USR) in a positive experience that far exceeded the expectations projected, to the benefit of the educational quality of broad sectors of the country.
12	AC12	Hinojosa, C., Hernández, M., Polo, B., y Morante, M. (2022)	Responsabilidad social universitaria y el proceso formativo de estudiantes en Perú University social responsibility and the formative process of students in Peru	Quantitative descriptive study which analyzed the responses of 109 university teachers	Teachers should promote innovative teaching strategies, the usual change caused by the pandemic and to value the management of virtual tools associated with collective practices of responsibility and social protection.
13	AC13	Flores, L., Severino, P., Sarmiento, G., y Sánchez, J. (2022)	Responsabilidad social universitaria: diseño y validación de escala desde la perspectiva de los estudiantes de Perú *[University social responsibility: scale design and validation from the perspective of students in Peru]*	Quantitative descriptive study which analyzed the responses of 150 university students, who were submitted to a questionnaire on USR.	It concludes that the perception of USR of students in Peru is of enormous relevance because it allows the design of strategies, policies, programs and practices that can contribute to the needs of students, in addition to the problems that may exist in society.
14	AC14	Severino, P., Sarmiento, G., Villar, J., y Ramírez, R. (2022)	Consumo sustentable socialmente responsable: El caso de estudiantes universitarios de una ciudad de Perú *[Socially responsible sustainable consumption: The case of university students in a Peruvian city*]	Quantitative descriptive study which analyzed the responses of 318 university students.	It is concluded that it is necessary to promote through the substantive functions of universities the integration of socially responsible and environmentally conscious actions.
15	AC15	Condori, M., Villavicencio, A., y Reyna, G. (2022)	Responsabilidad social universitaria: Percepción de docentes y autoridades de universidades públicas peruanas *[University social responsibility: Perception of teachers and authorities of Peruvian public universities]*	Correlational study of mixed approach which analyzed 21 subjects who were submitted to a questionnaire and 5 people were interviewed.	The results show that the perception of university social responsibility held by teachers and authorities is positive in its dimensions: organizational management, training and social participation, while in the research dimension it is negative. The need to strengthen the functioning of public universities through the institutionalization of a social responsibility policy is evident.
16	AC16	La Cruz, O., Zelada, E., Aguirre, J., y Garro, L. (2022)	Responsabilidad social universitaria y posicionamiento de universidades en Lima-Perú *[University social responsibility and positioning of universities in Lima, Peru]*	Quantitative descriptive-correlational study which analyzed the responses of 607 university students on the perception of USR within their respective universities.	It concludes that USR is relevant for university institutions to achieve a better positioning, since, by contemplating aspects such as training, research, management and extension, it allows them to have a better identity, communication and image among their target public.
17	AC17	Monzón, A., Illa-Sihuincha, G., Ruiz Villavicencio, R., y Candia Menor, M. (2022).	Neuromanagement y responsabilidad social: Factores clave en la gestión educativa universitaria *[Neuromanagement and social responsibility: Key factors in university educational management]*	Quantitative correlational study which analyzed the responses of 150 employees of a Peruvian university on the perception of the USR within the workplace.	The results show that university social responsibility provides explanatory content to certain aspects of neuromanagement, and that it represents a fundamental tool that includes the development of emotional factors related to concern for society and the environment.
18	AC18	Condori, M., y Reyna, G. (2019)	Percepción de la responsabilidad social universitaria en estudiantes de la Facultad de Sociología de una universidad pública de la ciudad de Huancayo, Perú *[Perception of university social responsibility in students of the Faculty of Sociology of a public university in the city of Huancayo, Peru*]	Quantitative descriptive study which analyzed the responses of 212 university students on the perception of the USR within their house of study.	concluding that the students of the Faculty of Sociology perceive that university social responsibility at UNCP is deficient.
19	AC19	Albertini, J. (2015)	La vigencia de la responsabilidad social universitaria en el Perú: una propuesta desde el capital social *[The validity of university social responsibility in Peru: a proposal from the perspective of social capital]*	Mixed study where he surveyed several people belonging to private and public universities and also conducted an analysis of various documents regarding USR.	The author initially expresses his doubts about the use of the term "social responsibility" in the university context, considering it redundant in relation to the traditional "social projection". However, he recognizes that changes in universities, such as competition and commercialization, have affected their commitment to the community. Therefore, he understands the importance of promoting social responsibility in the universities.
20	AC20	Díaz, M., Castillo, R., y Criollo, V. (2022)	Responsabilidad social de la universidad peruana en el contexto de la educación virtual *[Social responsibility of Peruvian universities in the context of virtual education]*	The methodology used is a qualitative, documentary approach, using content analysis.	concludes that universities should assume more efficiently and responsibly the processes of teaching, research, as part of the USR, and extension in virtual modality, following the norms and procedures established in the context of the pandemic.

Table 4 revealed that 65% of the analyzed literature was quantitative in nature, 30% was qualitative, and only 5% was mixed methods.

**Table 4.** Methodology used by scientific journals.

Code	Methodology		
	Qualitative	Quantitative	Mixed methods
AC1	X		
AC2	X		
AC3	X		
AC4	X		
AC5	X		
AC6	X		
AC7	X		
AC8	X		
AC9	X		
AC10	X		
AC11	X		
AC12		X	
AC13			X
AC14		X	
AC15		X	
AC16		X	
AC17		X	
AC18		X	
AC19	X		
AC20	X		
Total	65%	30%	5%

Social responsibility is being widely addressed as it has become a fundamental part of university policy ( Martí, *et al.*, 2019). From its inception, it was included in the University Law 23733 which stated that “universities extend their educational activities in favor of those who are not their regular students; in this sense, they organize activities to promote and disseminate general culture and professional studies, which can be free or not, and which can lead to certification.” In this way, the law aimed to establish a university-society interrelationship. However, since this was often confused with carrying out social assistance activities or mere cultural activities, the new University Law 30220 was enacted, which incorporated university social responsibility and was regulated through the quality model of the National System for Evaluation, Accreditation, and Certification of Educational Quality (2019) [2016, 2017]. This quality model includes responsibility as part of institutional management, with Factor 6 focusing on responsibility. It states that “the university has a defined policy of social responsibility, and its actions are framed within it, achieving integration with research, technological development, and innovation activities (R&D&I)” ( Villegas and Cairampoma, 2021, p.3). This factor undoubtedly urges universities to involve faculty and students in social and environmental development projects and to engage the entire university community with stakeholders from the public and private sectors, thus contributing to society.

Furthermore, Article 125 of the law requires universities to allocate a minimum of 2% of their budget to social responsibility actions and provides instruments to incentivize the development of projects such as social responsibility initiatives or the establishment of competitive funds ( Villegas and Cairampoma, 2021).

In other words, university social responsibility concerns not only students but also other areas, including administrative functions such as purchasing, planning, service provision, enrollment, marketing, and personnel selection, among others. It also extends to the academic dimensions of education and research ( Vallaeys and Álvarez, 2019), as we must remember that the university becomes the center for knowledge production and professional training, with a significant impact on students’ lives ( Zolezzi, 2010). The university allows all its members to develop social, environmental, and economic awareness ( Severino, Sarmiento, Villar, and Ramírez, 2022).

Martí *et al. *(2018) conducted an analysis of university social responsibility in three countries, including Peru, where they found that it is implemented within the framework of the University Law 30220 (Ley 30220, 2014). They highlighted that the objective of university social responsibility is to generate mutual benefits between the university and the community, aiming to create development improvements that contribute to the well-being of citizens and the nation. In addition to being a requirement in the curriculum, university social responsibility serves as a support base for accreditation. It obliges higher education institutions (HEIs) to implement, develop, and put into practice social responsibility activities within their undergraduate programs. The goal is to prepare graduates who can contribute to and implementing “public policies of social interest and fostering altruistic and supportive behavior that contributes to improving the quality of life of vulnerable groups in our society” (Ley 30220, 2014, p.57), based on the knowledge and skills they have developed during their professional training.

Vargas (2021) presents a concept of university social responsibility from the perspective of innovation, indicating that every university should channel “the provision of a public service of knowledge generation where the general interests of citizens, the defense of the common good, and functioning as an agent of change and social improvement must be considered” (p. 442). In line with this, Ruiz (2018) discusses a proposal made by a university in collaboration with the Ministry of Education and three other universities, which involves granting a scholarship to students who have completed their undergraduate studies and wish to pursue a master’s degree within the same institution. This is known as the “Teaching Scholarship,” and its purpose is to provide social and economic support to professionals from any region of Peru, allowing them the opportunity to further their education and become master’s degree holders in their chosen field, with full academic preparation. The aim is to promote a culture of meritocracy within the teaching staff.

In 2017, the Universidad Los Ángeles de Chimbote implemented 3,716 social development projects, involving over 44,399 students and receiving guidance from 537 teachers. The target population benefited from these projects consisted of vulnerable communities in the region where the university is located. Similarly, the Pontifical Catholic University of Peru has made more concrete progress by engaging in activities across various dimensions such as the academic sphere, society, campus, strategic communication, and administrative strategies. Additionally, each organ, department, faculty, academic department, center, and institute within the university aims to strengthen the integration of this important topic in a comprehensive manner ( Villegas and Cairampoma, 2021).

Ortiz (2010) conducted a study on a specific course called “Proyección Social” (PROSODE) offered at the Pontifical Catholic University of Peru (PUCP) in the Law program. PROSODE is based on university social responsibility (USR), and the author emphasizes that the real purpose of USR is to act in the community and have this action benefit the university community as a source of learning. The course is designed for the development and execution of activities and projects for the benefit of citizens. Its objectives include exercising law in favor of those in need, generating experiences related to USR and being a lawyer, understanding the reality of Peru, and preparing students to fulfil their professional roles when the time comes. This perspective is supported by La Cruz, Zelada, Aguirre, and Garro (2022), whose study revealed a direct relationship between USR and four components: ‘Formation’, ‘Research’, ‘Management’, and ‘Extension’. The study concluded that USR is relevant for university institutions to achieve better positioning, as it encompasses aspects such as education, research, management, and extension. It allows for a stronger identity, communication, and image among the target audience. Therefore, policies and procedures should be in place to encourage greater participation of all university members in USR activities ( La Cruz *et al., *2022, p.341).

Hinojosa *et al. *(2022) also emphasize the importance of university social responsibility in the context of teaching and learning. According to these authors, it is necessary to promote a participatory dynamic in university classrooms, utilizing different methods and virtual tools, all from a perspective of social responsibility. They consider social responsibility as a crucial pillar in the theoretical and practical consolidation of teaching processes and aim to ensure that these practices reflect their own characteristics, adapting to any eventualities that may arise ( Hinojosa *et al., *2022, p.1094).

In summary, Kovács’s analysis (2016) highlights the importance of generating knowledge and findings based on the national reality, particularly in remote communities, while respecting their human dignity and cultures. It also emphasizes the need to eliminate the limited view of social responsibility as mere donations or sponsorships and instead promote the creation and management of knowledge for the benefit of society.

Díaz, Castillo, and Criollo (2022) point out that in 2020, with the arrival of COVID-19 in Peru, many higher education institutions (HEIs) had to close their doors and pause all scheduled activities for the start of the academic year due to the pandemic. This led to the suspension of in-person activities in universities and the need to implement contingency measures that would allow teaching to continue for students. The quickest solution was to transition to virtual teaching. This change posed a significant institutional challenge as it required guidance not only for students but also for faculty members. The new method of teaching had to maintain the same level of commitment and moral obligation as the classroom setting, ensuring the production of new knowledge relevant to solving citizens’ problems and the direct application of scientific and technological knowledge, as well as a more humanitarian professional preparation ( Díaz *et al.*, 2022, p.335). This means that the essence of university social responsibility should not be neglected. Therefore, “it is necessary for different universities to guarantee the same quality of education in the virtual context as in the in-person setting, which is part of this university social responsibility” ( Díaz *et al.*, 2022, p.336). This should be done while upholding the principles of university social responsibility as a social proposal, as expressed by Albertini (2015), who states that “universities can improve and enrich collaborative work experiences between the state and the population through co-production projects. They can provide guidance for the development of realistic participatory budgets and support their monitoring and proper implementation. They should raise the level of political and public debates by scrutinizing the suitability of proposals and demanding efficient and effective solutions for the problems faced by our communities and regions.”

Now all of this must also be seen from the student’s perspective, whether they perceived and felt that their academic preparation was humanistic and geared towards the country’s development. For this reason, a study was conducted by Pumacayo *et al. *(2020) to investigate the perceptions of USR among students. The study focused on a group of students from various faculties of a public university in Peru, aiming to understand their perception of USR and whether they believed that the university implemented it in their professional preparation. The results showed that 35.4% of students considered USR to be negative at the university, followed by 33.2% who rated it as average, and only 8.2% rated it as very good.

In universities such as the Hermilio Valdizán National University, activities related to university social responsibility have been carried out through projects and/or plans of University Extension and Social Projection ( Saquicoray and Cuadros, 2015). However, since the concept of social responsibility is broader, some studies indicate that these efforts are not sufficient. According to a study conducted by Condori and Reyna (2019), 58% of students from the Faculty of Sociology at the National University of Central Peru still consider social responsibility to be deficient. This perception includes dimensions such as environmental management and governance, where they also believe that social participation is passive. Therefore, there is still a need to work diligently to strengthen the implementation of university social responsibility in public universities in the interior of the country. While effective social participation is already carried out through volunteer actions, university extension, and social projection, it is necessary to include aspects such as knowledge dissemination and transfer through research and sustainable organizational management ( Condori *et al.*, 2022).

Flores *et al. *(2022) questioned the constructs that would allow measuring USR from the perception of university students in Peru. To do this, they designed a rating scale to measure university social responsibility from the perspective of Peruvian students and obtained the following dimensions: “Broad and transformative academic education, socio-environmental engagement, socially focused research, and transformative institutional management and administration” ( Flores *et al., *2022, p.94). It is worth noting that this scale was validated and reviewed by experts in the field who approved the instrument.

As observed in Peru, the law regulates processes and encourages universities to promote challenges in the education of their professionals, making them socially responsible, useful, and capable of promoting actions that contribute to equality, societal well-being, and sustainable development. According to Severino *et al. *(2022), university social responsibility incorporates the dimension of sustainable consumption as part of its improvement processes. It can be understood that a socially responsible university student exercises their consumption in a sustainable manner. This implies that the responsibility exercised by the university has a transformative capacity and is a fundamental axis for the implementation of educational policies in different higher education institutions. Therefore, it is essential to seek qualities in teachers, such as mental and emotional management of guiding environments. They should be able to share motivations, achieve the objectives of proposed plans, and improve educational productivity, thus reinforcing the practical dimension of individuals not only through the curriculum but also in other areas such as management, research, and university outreach to society ( Monzón *et al., *2022).

## Discussion

Within the literature found, two points are quite clear when discussing university social responsibility. Firstly, the main beneficiary should always be society, as it is where university students coexist, and universities have the fundamental task of implementing and constantly developing USR within their institutions, ensuring its practical application among all members. This is expressed by Ruiz and López (2019) when they state that “the university cannot remain detached from the reality in which it exists; it must be a driver of development and social cohesion.” This should be achieved through various fields, not only through research but also through humanistic, social, and technological practices. Implementing this will provide students with education in principles and ethics, which will positively impact their professional activities and their contribution to society. Bracamonte and Reynafarje (2017) support this by concluding that USR within universities should be oriented towards the commitment that students, teachers, and authorities must have in contributing to the development and formation of a better society.

Although most of the studies included in this review agree in highlighting the importance of USR as a transformative function of the university, critical approaches and divergent points of view were also identified. For example, Albertini (2015) argues that the concept of USR can be redundant or superficial if it is not clearly differentiated from the traditional university social projection. From this perspective, the use of the term USR could be perceived as a discursive adaptation rather than a structural transformation.

Likewise, Zolezzi (2010) warns that many universities, especially private ones, prioritize business training to the detriment of social commitments. This critical view suggests that the implementation of USR is often limited to isolated actions, with no real impact on ethical training or the transformation of the environment. Similarly, studies such as those by Condori and Reyna (2019) show a negative or deficient perception on the part of the student body, which indicates a disconnection between the institutional discourse on USR and its concrete application.

These perspectives highlight the need to critically review university practices and move towards models of USR that are not limited to voluntary or welfare activities, but are deeply articulated with the curriculum, applied research and sustainable development.

It is important to mention that this analysis could have limitations, such as the lack of diversity in the bibliographic sources consulted or the lack of empirical support for the assertions made. In addition, the literature review may be subject to selection biases and limitations in the completeness of the search for sources. It should also be kept in mind that the interpretation of the results may be influenced by the author’s biases or theoretical approaches.

The results have implications relevant to practice, policy, and future research. They support the need for universities to assume university social responsibility as a central aspect, developing programs and practices that foster the engagement of students, faculty and authorities in contributing to social development. This implies practical considerations for the design of curricula, extension programs and university policies. It also suggests areas for future research to better understand the impacts and best practices of university social responsibility in the academic context.

## Conclusion

Within the Peruvian context, it is evident that there is still much work to be done, as the student body does not view the promotion of USR within their educational institutions favourably, indicating that they do not feel their academic preparation is being carried out with a responsible focus that benefits society. Pérez (2020) supports this by finding that the perception level of students in a university in Peru regarding university social responsibility was low. This is often because teachers and/or authorities do not encourage it within their educational institutions. Híjar (2018) found that “university authorities interviewed indicated that there are several aspects that can be improved regarding university social responsibility within the educational institution. However, they do not specify what the university is doing to improve it,” and the teaching staff had a moderate perception of USR.

Therefore, there is a need to create additional policies and procedures that encourage greater participation from all members of the university in USR activities, rather than isolated cases in some universities, as evidenced in certain studies. It is worth noting that with the arrival of COVID-19 and the new forms of virtual teaching, it should not be a reason to give up and neglect USR. On the contrary, the necessary conditions must be created for universities to engage in more innovative work, which, in turn, meets the expectations of the community and the country regarding their university graduates.

This systematic review provides a robust analysis of USR in Peru, addressing critical methodological and policy-related gaps. By integrating a comprehensive evaluation of existing literature, discussing practical implications, and incorporating diverse perspectives, this study enhances the discourse on USR and its role in shaping responsible professionals. Future research should focus on longitudinal studies that assess the tangible outcomes of USR initiatives in diverse educational settings.

## Data Availability

Zenodo. University Social Responsibility (USR) in the context of Peruvian society A systematic review. Version 3. https://zenodo.org/record/8190897 ( Arbieto *et al., *2023). This project contains the following underlying data:
• Manuscripts analyzed.xlsx. (Final manuscripts included in the systematic review). • Zenodo: PRISMA checklist for ‘University Social Responsibility (USR) in the context of Peruvian society A systematic review’. https://doi.org/10.5281/zenodo.8190897 ( Arbieto *et al., *2023). Data are available under the terms of the Creative Commons Attribution 4.0 International license (CC-BY 4.0).
