# Supplementary material for: University social responsibility (USR) in the context of Peruvian society: A systematic review
Source: F1000Res. 2026 Mar 4;12:1170. Originally published 2023 Sep 19. [Version 3] doi: 10.12688/f1000research.138153.3 (PMC12972712; doi:10.12688/f1000research.138153.3)
Supplement: Peer review history

# Reviewer report: Reviewer response for version 3

**DOI:** 10.5256/f1000research.196917.r465298

**Published:** 2026-03-18

**Authors:** Bernardo Nahuat Román

**Affiliations:** 1 Universidad Autonoma de Tamaulipas, Ciudad Victoria, Tamaulipas, Mexico

**General evaluation**

 The article addresses an interesting and timely topic that has been gaining increasing attention in the academic literature. The study has potential; however, several adjustments are recommended in order to strengthen its methodological rigor, clarity, and overall contribution to the field.

**Abstract**

- In the *background* section, I suggest avoiding the repetition of the expression “goes beyond”, which appears twice. Using synonyms would improve the readability and flow of the text.

**Introduction**

- The authors should provide a stronger justification for focusing on Peru as the context of the study. It would be helpful to explain why Peru represents a relevant case for analyzing University Social Responsibility and what characteristics of the Peruvian higher education system make this review particularly pertinent.

**Method**

- I recommend including the Web of Science database in the search strategy. This is one of the most important databases for systematic reviews, and its exclusion should either be justified or reconsidered.
- The section should avoid redundancies in the methodological description. For example, the PRISMA methodology is mentioned repeatedly in consecutive paragraphs.
- The authors indicate that “Peru” was used as one of the search terms. However, it is unclear how studies were treated if they focused on Peruvian universities but used the name of a specific city or region instead of “Peru” in the title. Clarification or expansion of the search strategy would strengthen the methodological transparency.
- Although the review was originally conducted in 2023, the current year is 2026. Given that this is a growing research field, the authors should update the literature search to include more recent publications. Otherwise, the findings presented may already be partially outdated.

**Results**

- Although the number of articles included in the review is relatively small, the dataset could be analyzed in greater depth. For instance, it would be useful to identify:
  - the most productive or most cited authors.
  - the most frequently used theoretical perspectives.
  - the dimensions of University Social Responsibility most commonly addressed in the literature.
  - emerging trends or research gaps.
- Whenever possible, quantitative information should be provided. Expressions such as “many” or “few” are ambiguous and could be replaced with numbers or percentages.
- Redundancies in the presentation of the results should be avoided.
- It is not clear why Table 3 includes a citation in its title. This should be reviewed and, if necessary, the citation could be placed as a note instead.
- Some paragraphs appear to belong more appropriately to the theoretical framework rather than the results section (for example, the text on page 11). The structure of the manuscript should be reviewed to ensure clear separation between sections.
- In this section, the focus should be on synthesizing the main findings of the reviewed literature, rather than providing extended descriptions of individual articles.

**Discussion**

- The discussion would benefit from a clearer explanation of which perspectives have been widely explored, and which remain underexplored, particularly in comparison with other literature reviews on the topic.
- It would also be valuable to contextualize the findings by comparing them with similar studies conducted in other Latin American countries.
- Some paragraphs in this section appear to correspond more closely to concluding remarks, so the distinction between discussion and conclusions should be clarified.

**Conclusions**

- The study includes only 20 articles, and the literature search was conducted in 2023, which limits the scope of the conclusions. These aspects should be explicitly acknowledged as limitations of the study.

**Overall comments**

- The manuscript would benefit from updating the literature throughout the entire document, incorporating more recent studies on University Social Responsibility.
- In its current form, the article appears to be primarily descriptive, which limits its contribution to the advancement of knowledge in this field. Strengthening the analytical and critical discussion, as well as identifying clear research gaps, would significantly enhance the manuscript.

Are the rationale for, and objectives of, the Systematic Review clearly stated?

Yes

Is the statistical analysis and its interpretation appropriate?

Partly

If this is a Living Systematic Review, is the ‘living’ method appropriate and is the search schedule clearly defined and justified? (‘Living Systematic Review’ or a variation of this term should be included in the title.)

Not applicable

Are sufficient details of the methods and analysis provided to allow replication by others?

Partly

Are the conclusions drawn adequately supported by the results presented in the review?

Partly

Reviewer Expertise:

Corporate Social Responsibility, University Social Responsibility, Innovation

I confirm that I have read this submission and believe that I have an appropriate level of expertise to confirm that it is of an acceptable scientific standard, however I have significant reservations, as outlined above.

# Reviewer report: Reviewer response for version 3

**DOI:** 10.5256/f1000research.196917.r465296

**Published:** 2026-03-09

**Authors:** Sri Suranta

**Affiliations:** 1 Universitas Sebelas Maret, Surakarta, Indonesia

Reviewer Comments:

 General Comments:

 This article provides a specific contribution to Peruvian society regarding the importance of universities' social responsibility to society and the potential for developing ethical and responsible students.

 Specific Comments:

1. Define the Research Question. The research question is not explicitly stated and should be added in the introduction. For example, it can use frameworks like PICO (Population, Intervention, Comparison, Outcome) or SPIDER (Sample, Phenomenon of Interest, Design, Evaluation, Research Type) to structure your question. Example (PICO): How does mindfulness-based therapy (Intervention) compare to traditional cognitive behavioral therapy (Comparison) in reducing anxiety (Outcome) among adults (Population)?
2. The methods and analysis provided to allow replication by others. The methodology section, specifically the database search, does not specify the duration of the data search. This is because the start of the data search has not been explicitly stated, although the end of the search has been limited to February 2023. This needs to be clarified, whether the data collection began in 2014, when University Law 30220 (2014) came into effect, or from the previous year. 
3. Conclusion. The research conclusion refers less to the results and discussion, the conclusions stated are more about the practical implications of the research results. 

Are the rationale for, and objectives of, the Systematic Review clearly stated?

Yes

Is the statistical analysis and its interpretation appropriate?

Partly

If this is a Living Systematic Review, is the ‘living’ method appropriate and is the search schedule clearly defined and justified? (‘Living Systematic Review’ or a variation of this term should be included in the title.)

Yes

Are sufficient details of the methods and analysis provided to allow replication by others?

Partly

Are the conclusions drawn adequately supported by the results presented in the review?

No

Reviewer Expertise:

I am a researcher and lecturer at Sebelas Maret University, Surakarta, Indonesia. My research interests include accounting, taxation, and social responsibility, particularly in higher education.

I confirm that I have read this submission and believe that I have an appropriate level of expertise to confirm that it is of an acceptable scientific standard, however I have significant reservations, as outlined above.

# Reviewer report: Reviewer response for version 2

**DOI:** 10.5256/f1000research.180054.r449658

**Published:** 2026-01-22

**Authors:** Stephen T. Homer

**Affiliations:** 1 Sunway University, Sunway, Malaysia

***Abstract***

 The line ‘but the professional must improve and develop proposals in improvement of the country’ is unclear, with reference to the graduates or the academics?

 What is the modification behind the study? The problem it is trying to address?

 February 2023? It is now January 2026? Why such a gap?

 How can an indepth analysis be conducted with only 20 articles.

***Introduction***

 The opening is completely off topic, this reads more like a literature review, where you have start broad and then narrowed to the specific topic you are discussing. It is also quite sparce, would suggest breaking into Introduction section that is focused and in the format below, whilst also having a background section which shows the development you currently have written and elaborate upon both;

- Introduce your topic
- Describe the background
- Establish your research problem
- Specify your objective(s)
- Map out your paper

***Methods***

 What was the explicit Booleen phrase(s) used for the search?

 20 articles, since the databases were started? AS I don’t see a specific data range, this raises concerns of how to draw meaningful conclusions from such date dispersed literature as the phenomenon in question is dynamic and has changed drastically over recent years. A greater detail and defense of the choice made here need to be elaborated upon.

***Results***

 Having a table listing all 20 articles with some broad details, is just descriptive and take 4 whole pages. The table should be a summary if it is retained, with this large table being in the appendix as it adds very little to the study.

 Much of this appears to be descriptive and adds little to the body of knowledge.

***Discussion***

 This is the opportunity to show what you paper is contributing but it is very lacking in interpretation.

 The Discussion section should begin with a general discussion putting what the results mean in general language and this should be linked back to the prior literature, identifying where other authors have agreed or disagreed with your findings. Effectively summating what is different or the same about your findings for your specific study in Peru.

 Next should be Theoretical Contributions, I would recommend looking at Homer, S. T., & Lim, W. M. (2024). Theory development in a globalized world: Bridging “doing as the romans do” with “understanding why the romans do it”. Global Business and Organizational Excellence, 43(3), 127-138. This will help you position your study and how you can develop the theory, which should be introduced what theory is underpinning the study in the literature review, hence the importance when highlight a conceptual or theoretical model.

 Thus, it is important to set a clear context of the messo level of Peru and what makes it unique, so you can add to the theory around a specific scope. Thus, rather than trying to add to the generalizable theory, you add your own small specific piece which many have unique elements that disagree with the core theory. What from the Peruvian perspective is USR? You can construct a model from your findings here

 Following this, should be Practical Recommendations, here you should discuss why practitioners should want to take note of and how they can actually implement this within their specific universities within Peru, and then what perhaps is missing or different from the international context. The more specific the better, so that the application is tangible. What is the potential? This may also link to the university ranking systems such as the sustainability rankings in QS and THE.

 This could be followed by Policy Recommendations, both at the organisational level of the universities and at the governmental level, what policies would encourage the implementation of your findings and how would it be of benefit? Of course, this would follow on from what the practical implications are that you have defined.

***Conclusion***

 Structure here seems fine but will need to be updated inline with changes throughout the manuscript.

 The topic is promising but needs extensive reworking to ensure the contribution to the literature is captured.

Are the rationale for, and objectives of, the Systematic Review clearly stated?

Partly

Is the statistical analysis and its interpretation appropriate?

Partly

If this is a Living Systematic Review, is the ‘living’ method appropriate and is the search schedule clearly defined and justified? (‘Living Systematic Review’ or a variation of this term should be included in the title.)

Not applicable

Are sufficient details of the methods and analysis provided to allow replication by others?

Partly

Are the conclusions drawn adequately supported by the results presented in the review?

No

Reviewer Expertise:

Sustainability, Youth, ESD, RME

I confirm that I have read this submission and believe that I have an appropriate level of expertise to confirm that it is of an acceptable scientific standard, however I have significant reservations, as outlined above.

# Reviewer report: Reviewer response for version 2

**DOI:** 10.5256/f1000research.180054.r413354

**Published:** 2025-09-25

**Authors:** Moses Adeleke ADEOYE

**Affiliations:** 1 Al-Hikmah University, Ilorin, Nigeria

To make the article scientifically sound the authors **must** (1) supply reproducible search strategies for each database; (2) complete the PRISMA flow with explicit counts and exclusion reasons; (3) perform and report a formal quality assessment of included studies; (4) provide the data-extraction template / dataset; and (5) revise the synthesis and conclusions to reflect study quality and heterogeneity. Once these five items are addressed, the manuscript will meet essential standards for reproducibility and for appropriately evidence-based conclusions.

Are the rationale for, and objectives of, the Systematic Review clearly stated?

Yes

Is the statistical analysis and its interpretation appropriate?

Not applicable

If this is a Living Systematic Review, is the ‘living’ method appropriate and is the search schedule clearly defined and justified? (‘Living Systematic Review’ or a variation of this term should be included in the title.)

Not applicable

Are sufficient details of the methods and analysis provided to allow replication by others?

Partly

Are the conclusions drawn adequately supported by the results presented in the review?

Partly

Reviewer Expertise:

Educational Leadership, Research Methodology

I confirm that I have read this submission and believe that I have an appropriate level of expertise to state that I do not consider it to be of an acceptable scientific standard, for reasons outlined above.

# Reviewer report: Reviewer response for version 2

**DOI:** 10.5256/f1000research.180054.r399659

**Published:** 2025-08-26

**Authors:** David Perez Jorge

**Affiliations:** 1 Department of Didactics and Educational Research, University of La Laguna, La Laguna, Spain

I have reviewed your manuscript with great interest. The topic addressed is relevant and timely, as University Social Responsibility (USR) is a strategic axis for higher education, particularly in Latin America. The work integrates studies in both Spanish and English, follows PRISMA guidelines, and combines legislative, institutional, and empirical perspectives. These aspects, together with the identification of patterns in methodological approaches and thematic areas, strengthen the proposal.

 However, I believe the manuscript would benefit from major revisions to strengthen methodological transparency, update and expand the literature, and deepen the analytical synthesis of the findings. My main observations are as follows:

1. Theoretical framework and literature update: Incorporate more recent and international studies (last five years) that address conceptual frameworks, measurement methodologies, and comparative analyses of USR. Deepen the conceptual distinction between USR and traditional social outreach.
2. Methodology and transparency: Complete the search equations, including Boolean operators, truncations, and synonyms. Provide detailed reasons for exclusion at each stage of the PRISMA diagram. Clarify whether grey literature was considered and justify its inclusion or exclusion. Specify the data extraction and coding process. Include a formal quality assessment of the studies (e.g., CASP, MMAT).
3. PRISMA flow diagram: Update the PRISMA flow diagram to clearly specify:
  - The number of articles retrieved from each database.
  - The number of articles excluded based on each exclusion criterion.
    Currently, the information in the flow diagram is incomplete and hinders the traceability of the selection process.
4. Study synthesis table: Expand the synthesis table to include fundamental dimensions of analysis for the selected studies, such as:
  - Source quality.
  - Possible biases.
  - Sample size and characteristics.
  - Other relevant methodological aspects that allow the assessment of each study’s robustness.
5. Analysis and synthesis: Move from a mainly descriptive synthesis to a more comparative and structured analysis, incorporating thematic mapping or bibliometric analysis. Analyse the implications of the predominance of quantitative methodologies and systematise the presentation of contradictory findings.
6. Ethical aspects: Include a statement confirming that no primary data were collected and that all sources were used in compliance with copyright and licensing requirements.
7. Discussion and conclusions: Strengthen the link between findings and practical implications, with concrete proposals for institutional policies and curriculum design. Make recommendations for future research more specific. Reformulate conclusions with a greater focus on impact.
8. Limitations: Expand the reflection on potential biases, including restrictions in the databases consulted and the absence of longitudinal evidence.

In summary, the manuscript presents a valuable contribution to the field but requires these adjustments to reach the level of rigour and clarity expected in systematic reviews. I remain available to clarify any additional aspects you may require.

Are the rationale for, and objectives of, the Systematic Review clearly stated?

Yes

Is the statistical analysis and its interpretation appropriate?

Not applicable

Are sufficient details of the methods and analysis provided to allow replication by others?

Partly

Are the conclusions drawn adequately supported by the results presented in the review?

Partly

Reviewer Expertise:

Educational research, Special Education Needs, health education

I confirm that I have read this submission and believe that I have an appropriate level of expertise to state that I do not consider it to be of an acceptable scientific standard, for reasons outlined above.

# Reviewer report: Reviewer response for version 1

**DOI:** 10.5256/f1000research.151333.r239392

**Published:** 2024-02-14

**Authors:** David Perez Jorge

**Affiliations:** 1 Department of Didactics and Educational Research, University of La Laguna, La Laguna, Spain

I have had the opportunity to review your article entitled "University social responsibility (USR) in the context of Peruvian society: A systematic review". I am pleased to note that your work represents a significant effort in synthesizing research on a topic of increasing importance. However, I would like to suggest some areas for improvement that could further enrich your study.

1. Critical Evaluation of Studies: A deeper evaluation of the methodological quality and limitations of the included studies could offer a richer and more contextualized perspective of the findings.
2. Practical and Theoretical Implications: A more detailed discussion on how your findings can influence educational policies and practices would enrich your contribution to the field.
3. Data Analysis Methodology: Providing more details on the techniques used for data analysis would strengthen the transparency and replicability of your study.
4. Continuous Update and Review: Including recent studies and periodically updating the article would ensure that your review continually reflects trends and changes in the field.
5. Diverse Perspectives: Including studies with diverse or contradictory perspectives could provide a more comprehensive view of the topic.

I hope you find these comments useful for strengthening your work. I appreciate the opportunity to review your valuable research.

Are the rationale for, and objectives of, the Systematic Review clearly stated?

Yes

Is the statistical analysis and its interpretation appropriate?

Not applicable

Are sufficient details of the methods and analysis provided to allow replication by others?

Partly

Are the conclusions drawn adequately supported by the results presented in the review?

Partly

Reviewer Expertise:

Educational research, Special Education Needs, health education

I confirm that I have read this submission and believe that I have an appropriate level of expertise to confirm that it is of an acceptable scientific standard, however I have significant reservations, as outlined above.

# Reviewer report: Reviewer response for version 1

**DOI:** 10.5256/f1000research.151333.r218915

**Published:** 2023-11-30

**Authors:** Edgard Méndez

**Affiliations:** 1 Antenor Orrego Private University, Trujillo, La Libertad, Peru

Interesting article developed, I highlight the results that could be found from the reviews of the works.

 The systematic review on USR in Peru found that there is still much work to be done in promoting USR within educational institutions, as the student body does not view it favorably. This is often because teachers and/or authorities do not encourage it within their educational institutions. The review also highlights the need to create additional policies and procedures that encourage greater participation from all members of the university in USR activities, rather than isolated cases in some universities.

Are the rationale for, and objectives of, the Systematic Review clearly stated?

Yes

Is the statistical analysis and its interpretation appropriate?

Yes

Are sufficient details of the methods and analysis provided to allow replication by others?

Yes

Are the conclusions drawn adequately supported by the results presented in the review?

Yes

Reviewer Expertise:

Business Administrator

I confirm that I have read this submission and believe that I have an appropriate level of expertise to confirm that it is of an acceptable scientific standard.
